# Alternative Vascularization Mechanisms in Tumor Resistance to Therapy

**DOI:** 10.3390/cancers13081912

**Published:** 2021-04-15

**Authors:** Dorina Belotti, Denise Pinessi, Giulia Taraboletti

**Affiliations:** Laboratory of Tumor Microenvironment, Department of Oncology, Istituto di Ricerche Farmacologiche Mario Negri IRCCS, 24126 Bergamo, Italy; denise.pinessi@marionegri.it

**Keywords:** tumor vasculature, antiangiogenic therapy, drug resistance, non-sprouting angiogenesis, tumor microenvironment

## Abstract

**Simple Summary:**

Tumors rely on blood vessels to grow and metastasize. Malignant tumors can employ different strategies to create a functional vascular network. Tumor cells can use normal processes of vessel formation but can also employ cancer-specific mechanisms, by co-opting normal vessels present in tissues or by turning themselves into vascular cells. These different types of tumor vessels have specific molecular and functional characteristics that profoundly affect tumor behavior and response to therapies, including drugs targeting the tumor vasculature (antiangiogenic therapies). In this review, we discuss how vessels formed by different mechanisms affect the intrinsic sensitivity of tumors to therapy and, on the other hand, how therapies can affect tumor vessel formation, leading to resistance to drugs, cancer recurrence, and treatment failure. Potential strategies to avoid vessel-mediated resistance to antineoplastic therapies will be discussed.

**Abstract:**

Blood vessels in tumors are formed through a variety of different mechanisms, each generating vessels with peculiar structural, molecular, and functional properties. This heterogeneity has a major impact on tumor response or resistance to antineoplastic therapies and is now emerging as a promising target for strategies to prevent drug resistance and improve the distribution and efficacy of antineoplastic treatments. This review presents evidence of how different mechanisms of tumor vessel formation (vasculogenesis, glomeruloid proliferation, intussusceptive angiogenesis, vasculogenic mimicry, and vessel co-option) affect tumor responses to antiangiogenic and antineoplastic therapies, but also how therapies can promote alternative mechanisms of vessel formation, contributing to tumor recurrence, malignant progression, and acquired drug resistance. We discuss the possibility of tailoring treatment strategies to overcome vasculature-mediated drug resistance or to improve drug distribution and efficacy.

## 1. Introduction

During embryogenesis, primitive blood vessels are formed by vasculogenesis (differentiation from vascular precursors), followed by extensive new vessel formation from existing ones (sprouting and splitting angiogenesis) and subsequent vasculature maturation and remodeling. In adults, blood vessels are formed in a tightly controlled manner, in response to specific physiological requirements or as dynamic responses to pathological conditions.

The acquired ability to induce the formation of a functional vasculature is a hallmark of malignant cancer [1]. Vessels in tumors are heterogeneous, and are often immature, aberrant in morphology and function. They have discontinuous endothelium, basement membrane and pericyte coverage, high permeability, leakiness, and poor perfusion. Despite this, the vessels are necessary for tumor growth and metastasis, making them logical targets for therapies. However, preclinical and clinical evidence indicates that the hypoxic conditions induced by therapeutic vessel disruption, rather than constraining the tumor growth, actually activate a complex response that leads to alternative mechanisms of vessel formation and the recurrence of an aggressive and drug-resistant tumor.

Solid tumors employ various physiological mechanisms to generate a functional vasculature, from vasculogenesis to sprouting and non-sprouting angiogenesis. They may also use alternative, cancer-specific strategies, by exploiting existing vessels (vessel co-option) or by direct participation of the tumor cells themselves in the formation of vessels (vasculogenic mimicry and endothelial transdifferentiation). Different modalities of vessel formation often coexist in tumors, spatially and temporally regulated as a dynamic adaptation to environmental changes (hypoxia, growth factors, hemodynamic forces, or antineoplastic therapies), in a constantly evolving process of vessel generation and remodeling.

The connection between the tumor vasculature and drugs is multifaceted [2,3,4,5,6]. A two-way, mutual relationship between therapies and the vessels contributes to intrinsic or acquired resistance to antineoplastic therapies. As the main route of drug distribution, blood vessels’ functional properties—such as perfusion, flow, and permeability—control the diffusion of molecules to tissues. Therefore, the functional properties of existing vessels and the tumor microenvironment (TME) profoundly affect the distribution and efficacy of therapies. Endothelial cells in tumor vessels also regulate the extravasation of immune cells in tumors, hence determining tumor response to immunotherapies. In addition, vessels and molecular effectors of tumor vessel development can modulate cell responses to therapies through regulation of the metabolic and physical properties of the local environment. For example, hypoxia, acidosis, and high interstitial pressure deriving from vascular alterations lead to an immunosuppressive microenvironment that limits the efficacy of immunotherapy [7]. Finally, endothelial cells directly control chemosensitivity, through the release of angiocrine factors [8], contributing to tumor sensitivity or innate, intrinsic drug resistance.

On the other hand, therapies induce a process of vasculature and TME reprogramming that can sustain acquired drug resistance, tumor recurrence, and progression [2]. Vascular endothelial growth factors (VEGF) inhibitors are an example of this, as they are active on tumors relying on VEGF-dependent angiogenesis such as renal cancer (drug-sensitive), but not on tumors sustained by VEGF-independent forms of vessel formation (innate resistance) or tumors recurring after treatment, that present VEGF-independent compensatory programs of neovascularization (acquired resistance). Moreover, inhibitors of VEGF can prevent VEGF-induced immunosuppressive effects and potentiate the response to immune-checkpoint inhibitors and, vice versa, immunotherapies can promotes vascular changes and improve the efficacy of antiangiogenic therapies [9].

Rather than being determined by genetic or epigenetic events, microenvironment-mediated drug resistance is often the consequence of a shift in phenotype of the cells in the TME and in their interactions, as well as metabolic changes and alterations in the molecular and physical properties of TME components.

The importance of sprouting angiogenesis (SA) in tumor progression and response to antineoplastic drugs has been discussed in a number of comprehensive reviews [10,11,12,13,14] and will not be addressed here. Instead, this review will focus on other modes of vessel formation, including physiological processes (vasculogenesis, glomeruloid proliferation, and intussusceptive angiogenesis) and cancer-specific strategies (vasculogenic mimicry and vessel co-option). Attention will be given to the evidence that these modalities represent an adaptation to the pressure of therapies, and to their important contribution to tumor response/resistance to antiangiogenic and antineoplastic therapies.

## 2. Vasculogenesis

**Figure 1:**
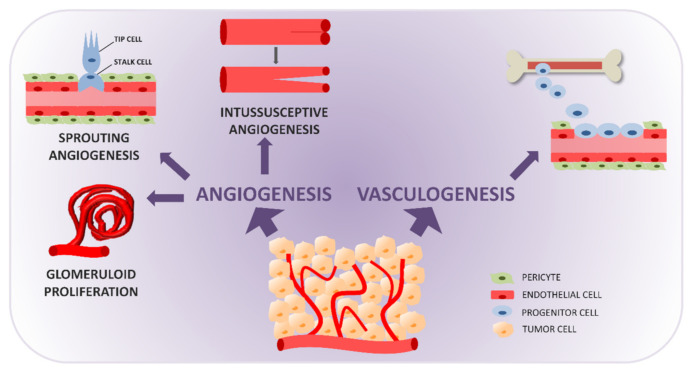
Formation of blood vessels in a tumor through physiological mechanisms: vasculogenesis and angiogenesis. Vasculogenesis is the de novo formation of vessels from bone marrow-derived precursors recruited to the tumor site. Angiogenesis is the formation of vessels from existing ones, through the processes of sprouting angiogenesis (endothelial cell specification into tip and stalk cells generate sprouts that elongate and fuse into new functional vessels), glomeruloid microvascular proliferation (deregulated proliferation of vascular endothelial cells generating glomeruloid structures), and intussusceptive angiogenesis (longitudinal splitting of vessels caused by the formation and extension of intra-luminar tissue pillars).

Vasculogenesis (Figure 1) is the de novo formation of vessels from precursor cells mobilized from the bone marrow, that enter the circulation and migrate to the site of vessel formation [14,15,16]. It typically occurs in the embryo, and post-natal vasculogenesis is considered a sporadic event, restricted to sites of physiological revascularization, injury repair, and vessel formation in pathological conditions.

Tumors can recruit vascular precursor cells, including endothelial progenitor cells (EPC), from bone marrow and also from adjacent tissues or circulating cells [16]. Stimulated by cytokines and growth factors produced by the tumor cells, mobilized precursor cells enter the circulation and are recruited to the tumor site, where they can be found associated to the vasculature. Here, precursor cells contribute to vessel formation by differentiating into vascular cells or by acting in a paracrine manner to promote vessel formation, although their actual role and relevance in tumor vessel formation still need to be clarified [14,17,18,19].

Despite initial difficulties in the definition of the phenotype of vascular precursor cells and differences in technologies to analyze and isolate them, a number of clinical studies have demonstrated the presence of circulating EPC in patients with hematological malignancies and solid tumors [15,20].

### 2.1. Mechanisms of Vasculogenesis

Hypoxia is a major trigger of tumor vasculogenesis. Hypoxia inducible factor (HIF)-1 stimulates the local production of factors, including stromal cell-derived factor 1 (SDF1, also known as C-X-C motif chemokine 12, (CXCL12) which promotes the recruitment of bone marrow EPC and CD45+ myeloid cells to the tumor site [21,22]. Hypoxia also stimulates the production of VEGF, reported to act by recruiting VEGF receptor 1 (VEGFR)1- and VEGFR2-expressing bone marrow EPC to the tumor site [23] or by potentiating the vasculogenic effect of SDF-1 [22]. Another factor important for vasculogenesis is matrix metalloproteinase-9 (MMP9), a downstream factor of HIF1α produced by recruited myeloid cells and tumor-associated inflammatory and stroma cells. MMP9 enables the recruitment of EPC to the tumor vasculature by increasing VEGF mobilization and bioavailability [21,24].

### 2.2. Vasculogenesis and Resistance to Therapies

Antineoplastic therapies, and particularly radiotherapy, can induce the mobilization of pro-angiogenic cells from the bone marrow, to sustain vasculogenesis and tumor recurrence. In most cases, this is linked to therapy-induced hypoxia at the tumor site. The effector of hypoxia HIF-1 stimulates the production of SDF-1, which recruits CXCR4-expressing cells, primarily CD11b+ myeloid cells, to the irradiated primary tumor [25].

The exact contribution of recruited bone marrow-derived cells to tumor re-vascularization after radiotherapy is not completely clear, although it appears that paracrine stimulatory effects on the surviving vessels might be more important than their direct differentiation into endothelial cells [26].

Recruited myeloid cells have also been implicated in the resistance to anti-VEGF and immunotherapies, potentially contributing to an angiogenic-to-vasculogenic switch, as well as to the generation of an immunosuppressive environment in recurrent tumors [2,27].

Cytotoxic drugs such as paclitaxel have been reported to inhibit angiogenesis [28], particularly as metronomic therapy, through the upregulation of thrombospondin-1 (TSP-1) [29]. However, cytotoxic drugs also induce systemic mobilization of bone marrow-derived proangiogenic and precursor cells which home to the tumor vasculature and sustain tumor-promoting vessel formation [30]. A similar induction of EPC mobilization and homing to tumors has been reported with vascular disrupting agents [31], confirming the complex role of therapies in shaping the TME.

### 2.3. Targeting Vasculogenesis

A number of compounds that can interfere with the vasculogenic process have been identified (reviewed in [27]), although in many cases, they cannot be considered specific inhibitors of the process, as they target factors with broad activities.

Inhibitors of the SDF-1 receptor CXCR4, such as AMD3100 (Plerixafor), prevent SDF-1/CXCL12-mediated recruitment of bone marrow-derived precursor cells to the tumor site [22,32]. When used shortly after local irradiation, AMD3100 successfully prevented the recruitment of vascular precursors, but it became ineffective if given later [32]. This suggests that therapies designed to prevent vascular regrowth and tumor recurrence after radiotherapy need to respect a strict therapeutic time window for effective intervention.

## 3. Glomeruloid Microvascular Proliferation

Angiogenic factor-induced vessel formation can give rise to different types of vessels, representing the progressive phases or different outcomes of evolution and maturation. Dvorak described at least six vessel types induced by VEGF, including earl-forming mother vessels and glomeruloid microvascular proliferations (GMP) and late-forming vascular malformations, capillaries, feeding arteries, and draining veins [33]. Although all these vessels were induced by VEGF, anti-VEGF/VEGFR therapy was effective only on early-forming GMP and mother vessels [34], suggesting that the different vessels can determine the sensitivity or resistance to antiangiogenic drugs.

GMP (Figure 1) is defined as endothelial cells arranged in multilayers, reminiscent of glomeruli, with a substantial amount of pericytes and macrophages. The relation between SA and GMP—both VEGF-dependent—is not completely clear. However, a recent study on brain vascular malformations indicated that a genetic defect in endothelial cells (specifically mutations in FLVCR2) impaired SA while causing GMP, suggesting substantial differences in the molecular requirements of the two processes [35].

Analysis of clinical samples indicated that GMP is a defining histological trait of glioblastoma multiforme [36], and has been found in other tumors, such as neuroblastoma [37], as well as in breast cancer, where it was associated with p53 overexpression, BRCA1 germline mutations, and other markers of aggressive disease [38,39,40]. GMP has been proposed as a marker of poor prognosis in patients with melanomas, breast, endometrial, and prostate cancer [38,41].

### 3.1. Mechanisms of GMP

VEGF is a major inducer of GMP [42]. A recent study implicated fibulin 7 (Fbln7)—highly expressed in endothelial cells and pericytes—in the formation of these vascular structures in glioblastoma. VEGF induces overexpression of Fbln7, that binds to pericyte-derived Ang1 and blocks the vessel-stabilizing Ang1-Tie2 signaling in endothelial cells. This might favor Ang2-Tie2 signaling, leading to the formation of GMP [43]. In addition, transforming growth factor-beta (TGFβ) signaling has also been identified as a key factor in regulating the vascular phenotype and GMP in glioblastoma [44].

### 3.2. GMP and Resistance to Therapies

GMP has not been associated with resistance to VEGF-targeting therapies. However, in line with the association of GMP with aggressive disease in breast cancer [31,32,33,34], high GMP was indicative of a poor clinical response to chemotherapy and reduced survival in breast cancer patients [31,32].

### 3.3. Targeting GMP

GMP is highly VEGF-dependent as it is inhibited by VEGF/VEGFR targeting therapies, including the VEGF trap Aflibercept [34]. Clinical studies confirm that the VEGFR inhibitor cediranib reduced the number of GMP in glioblastoma patients, although recurrent tumors presented evidence of vessel co-option [45]. Moreover, GMP in tumors was associated with objective responses to bevacizumab monotherapy in patients with metastatic melanoma [46]. At variance with these findings, high baseline GMP did not predict the response to bevacizumab in combination with chemotherapy in patients with advanced breast cancer, but was associated with markers of aggressive disease, including high histologic grade, basal-like, and triple-negative phenotypes [40]. This suggests a possible difference in the roles of these vascular structures in tumor progression and drug response, depending on the tumor type.

## 4. Intussusceptive Angiogenesis

Intussusceptive angiogenesis (IA) is the main alternative to SA in postnatal vessel formation and remodeling. IA is driven by physiological or pathological perturbations of vascular homeostasis, such as exercise, injury, aging, pharmacological intervention, or acute and chronic pathological conditions such as cancer [47,48]. IA (Figure 1) involves the longitudinal partitioning/splitting of an existing vessel, created by the insertion of intra-luminar pillars, that leads to the formation of two lumens and splits the original vessel into two new functional vessels [48,49,50]. It can take different forms: intussusceptive microvascular growth, arborization, and branching/remodeling. IA is a rapid adaptation of the vasculature to microenvironmental changes. Generation of vessels by IA does not necessarily depend on endothelial cell proliferation. It is fast, metabolically undemanding, and rapidly increases the number of vessels without altering permeability. IA has been reported to be more efficacious than SA in ensuring high oxygen levels [51].

IA has been observed in several solid tumors, in primary lesions, and metastasis, including experimental models of colon adenocarcinoma, melanoma, oral squamous cell carcinoma, and brain tumors [50,52,53,54].

IA is attracting increasing interest as a key process of vessel remodeling, and a valuable therapeutic target for tumors resistant to antiangiogenic therapy. However, IA studies have been hampered by a lack of experimental models and quantitative tools to identify and measure it in vivo [55]. Morphological examination of IA is usually based on the detection of intra-luminar pillars, the hallmark of IA, as holes in the corrosion casts or 3D reconstructions of the tumor vasculature, by electron microscopy or micro-CT analyses. New technological approaches are becoming available [56,57], hopefully contributing to a clearer definition of IA’s role in tumors and its potential as a therapeutic target.

### 4.1. Mechanisms of IA

IA has been mostly investigated in non-neoplastic settings, and, in many cases, the relevance of mechanistic insights derived from other conditions still needs to be verified in tumors.

Hemodynamic forces, changes in blood flow and shear stress, and oxygen consumption are considered major factors initiating the process of IA [47,58]. Several molecular pathways have been reported to be involved, including some master regulators of SA. Besides its main activity as an inducer of SA, VEGF has been involved in the initial phases of IA in skeletal muscle, depending on the ephrinB2/EphB4 axis. The interaction between ephrinB2 expressed by platelet-derived growth factor BB (PDGF-BB)-recruited pericytes [59] and endothelial EphB4 controls VEGF stimulatory effect on endothelial proliferation through ERK1/2 modulation. This limits vessel enlargement and enables vessel splitting while preventing VEGF-induced aberrant angiogenesis [56].

Endothelial cell expression of EphB4 is upregulated by Notch1 deletion, which promotes vessel remodeling and IA in liver sinusoidal vessels [60]. Notch inhibition also affects another pathway relevant for IA, the SDF-1/CXCR4 axis. SDF-1/CXCR4 signaling promotes IA in liver sinusoidal vessels through the recruitment of CXCR4+ and Tie2+ bone marrow-derived mononuclear cells, which have a stabilizing role in the formation of pillars [61]. SDF-1 expression in liver endothelial cells is controlled by Notch1, and inhibition of Notch signaling triggers SDF-1-induced mononuclear cell recruitment and IA [62].

Nitric oxide (NO) signaling contributes to physiological IA in tumors [63]. It has recently been shown that, by controlling NO production in endothelial cells, an MT1-MMP/TSP-1/αvβ3 integrin-CD47 axis regulates IA in a model of inflammatory colitis, a chronic inflammatory bowel disease [64]. Proteolysis of TSP-1 by the metalloproteinase MT1-MMP releases a carboxy-terminal TSP-1 fragment containing the binding sites for CD47 and the integrin αvβ3, which induces NO production and IA. Perturbations of this axis affect NO production, impairing vasodilation and preventing IA [64].

The involvement of the same factors and cell types in IA, SA, and also vasculogenesis, reveals the complex, dynamic molecular and cellular interconnections in the subtle balance of endothelial cell homeostasis, normal, and aberrant angiogenesis. This suggests that SA, IA, and vasculogenesis are complementary aspects of a single dynamic and highly coordinated program of vasculature adaptation to microenvironmental changes [58,65].

### 4.2. IA and Resistance to Therapies

A switch from SA to IA has been reported as a major mechanism of revascularization and tumor recurrence after antiangiogenic or antineoplastic therapy. In line with the multiple complex roles of VEGF, inhibitors of VEGF have different outcomes on IA, depending on the tumor type and the original pattern of vascularization.

In Ewing’s sarcoma, a reduction in IA shortly after treatment with CT-322 (an adnectin inhibitor of VEGFR-2) resulted in vessel normalization [66]. In contrast, in a model of murine breast carcinoma, regrowth of tumors after short treatment with the VEGFR-2 inhibitor vatalanib or fractionated radiotherapy was accompanied by vascular expansion through IA, with the involvement of alpha-smooth muscle actin αSMA-positive cells. IA was detectable immediately after cessation of therapy and was followed later by a second wave of SA [67]. A similar pattern of angio-adaptation after vatalanib treatment was documented in a murine renal cell carcinoma model, with the appearance of large sinusoid-like vessels and transluminal pillars in the vascular casts [68].

The observation that vessels generated by IA have better functionalities (less permeability and more perfusion) led to the hypothesis that IA might be a general program of tumor adaptive response to antiangiogenic drugs and might actually contribute to the transient normalization window elicited by these drugs [69]. Further studies are needed to assess whether IA, besides its role as a compensatory angio-adaptation favoring tumor recurrence and resistance to therapy, might be an exploitable opportunity to improve delivery of therapy.

### 4.3. Targeting IA

Given the elusive nature of tumor IA and the limited knowledge of the molecular mechanisms governing this process, it is not surprising that therapies to target tumor IA have not attracted the same interest and successful efforts as SA. The lack of experimental models of IA has further hampered the search for IA inhibitors for tumor therapy. Although some compounds have been reported to prevent IA, such as the CXCR4 antagonist AMD3100, which targets SDF1/CXCR4 signaling [61], evidence on the efficacy of strategies to act on IA and their value in cancer therapy are far from conclusive.

## 5. Vessel Co-Option

**Figure 2:**
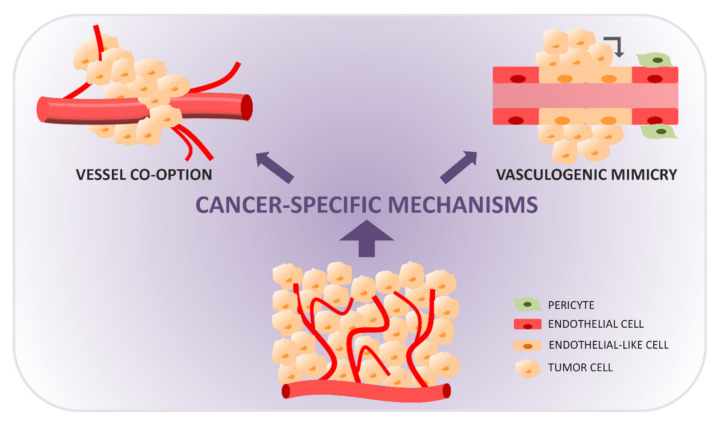
Tumor vasculature formation through non-angiogenic cancer-specific mechanisms: vessel co-option (cancer cells use pre-existing tissue blood vessels, migrating towards and along vessels and incorporating them into the tumor mass) and vasculogenic mimicry (cancer cells acquire endothelial-like properties and organize themselves in vascular-like functional structures connected to nearby blood vessels).

Vessel (or vascular) co-option (VC, Figure 2) is a mechanism by which tumors incorporate the existing vessels of the host organs, preserving the vascular scaffold of the surrounding tissues. In this way, a variety of tumors can grow without angiogenesis, obtaining nutrients and oxygen from vessels already present in the normal tissues and can grow infiltratively by migrating along them [70,71].

Co-opted vessels can be distinguished by histological analysis for the presence of distinct morphological features. Usually, they initially preserve the original architecture of the vessels of the host’s normal tissues with an intact basement membrane and mural cells. However, tumors hijacking vessels present an infiltrating growth pattern and can often remodel the original vasculature. Endothelial cells of co-opted vessels have a limited proliferation rate, distinguishing VC from SA better than microvascular density. Co-opted vessels poorly express markers of angiogenic endothelial cells such as αvβ3 in the lung and CD34 in the liver [48,71,72].

VC was first described in patients with lung cancers as an alveolar pattern of vascularization lacking parenchymal destruction, new-formed vessels, and tumor stroma [73]. This phenomenon has since been observed in various tumor types, especially brain, lung, and liver cancer, where both angiogenic and co-opted vessels often co-exist [70,74,75,76].

### 5.1. Mechanisms of VC

Co-opted vessels have been seen specially at the active proliferating edges of tumors, while angiogenic vessels tend to be located in the hypoxic center where HIF-target genes are activated. However, HIF activation does not always correspond to the activation of angiogenic pathways and does not necessarily lead to neovascularization but, depending on the genetic background of the neoplastic cells, it may lead to metabolic reprogramming. Non-angiogenic NSCLCs (non-small cell lung cancer) with co-opted vessels and angiogenic NSCLCs with respectively 70% and 32% of mutated p53 did not show a different expression of the genes usually associated with angiogenesis or hypoxia, but genes linked to metabolic reprogramming were expressed differently [77,78].

Another factor influencing VC is the interaction of tumor cells with the microenvironment of the organs where tumors develop or metastasize. NSCLC were classified as destructive, papillary, or alveolar depending on their growth pattern at the interface between the tumor and the normal lung, and their angiogenic profile. The alveolar subtype characterized by the presence of VC was associated with the absence of tumor-associated stroma at the invading edge and a low degree of endothelial cell proliferation [79].

In many tumors, the mechanism of vessel formation relies on both the intrinsic properties of tumors and on the characteristics of the organ microenvironment. Gliomas and glioblastomas offer an example of tumor plasticity and adaptation to the microenvironment [80]. These tumors use different vascular strategies including SA, vasculogenesis, trans-differentiation in endothelial cells, and VC depending on microenvironmental variability and response to treatments. A role for Wnt7 signaling in regulating single-cell co-option of blood vessels as opposed to collective perivascular migration has been described in mouse models [81].

Accordingly, vessels of primary tumors and metastasis may have different origins. Vascularization of metastasis is independent from the angiogenic potential of the primary tumors. Co-opted vessels are common in lung, liver, and brain metastasis, and in lymph nodes where metastatic cells exploit the well-vascularized microenvironment of the target organs to grow without stimulating new vessel formation [82,83,84,85].

### 5.2. VC and Resistance to Therapies

In the last few years, VC has received particular attention as a major mechanism of resistance to antiangiogenic treatment [86,87]. Besides being a characteristic of tumors intrinsically resistant to antiangiogenic treatment, VC may also be an adaptive mechanism to enable tumors to survive and progress when angiogenesis is inhibited [70]. Recurrent glioblastomas exploit VC as a strategy to escape anti-VEGFR treatments. These tumors initially respond to antiangiogenic therapy, but usually the benefits are transient. In brain autoptic samples from patients with recurrent glioblastoma treated with cediranib, there were structurally normal brain vessels, indicating co-option of the vessels [45].

Evidence of VC as a consequence of antiangiogenic treatments has also been reported in experimental models. Treatment with a neutralizing anti-VEGF antibody reduced tumor vascularization of human glioblastoma transplanted orthotopically in the basal ganglia of immunodeficient rats. However, after treatment, tumor cells deprived of nutrients and exposed to hypoxia migrated towards existing blood vessels [88]. Wnt7 signaling, mediating vessel co-option in gliomas, was upregulated by VEGF inhibitors (B20 and bevacizumab) and was proposed as a target to circumvent resistance to antiangiogenic therapies and to promote blood–brain barrier permeability and delivery of chemotherapy [81].

VC was also observed in colorectal cancer and breast cancer liver metastasis, where co-opted vessels were detected in patients who progressed after bevacizumab [89]. Similar results were obtained in hepatocellular carcinoma. In an orthotopic model of human hepatocellular carcinoma, sorafenib reduced SA while sparing vessels of the normal liver parenchyma. Normal vessels were then co-opted by the infiltrating tumor cells which undergo an epithelial-to-mesenchymal (EMT)-like transition and became invasive and resistant to the treatment [90,91].

### 5.3. Targeting VC

Since VC confers tumor resistance to antiangiogenic drugs, effective therapies should combine inhibition of both SA and VC. However, drugs specifically targeting VC have not been developed yet.

Intravital microscopy imaging and mathematical modeling have been used to analyze the dynamics of VC in glioma and brain metastasis during antiangiogenic treatments in mice. These models predicted that sequential targeting of co-option first, followed by VEGF inhibition, should give better results than simultaneous treatments [92]. Hypothetical targets were also identified using a theoretical model that analyzed the adaptive strategies of colorectal cancer liver metastasis, evaluating the role of each component of the microenvironment, such as immune cells, fibroblasts, and ECM, able to influence VC [93].

In the HT29 colorectal cancer model, cell motility through the actin-related proteins 2/3 (Arp2/3) complex proved essential for VC. Combined inhibition of Arp2/3 and angiogenesis was more effective in control HT29 liver metastasis than the single treatments [89].

Several pathways potentially involved in VC have been explored in glioblastoma. However, preclinical models to test their value as therapeutic targets are still needed. Promising targets include bradykinin, CXCR4/SDF1α, angiopoietin-2, IL8, EGFRvIII (a mutant isoform of EGFR), MDGI/FABP3, inositol-requiring enzyme (IRE)-1α, CDC42, and ephrin-B2 (reviewed in [80]).

Finally, since increased invasive behavior and EMT transition of tumor cells have been observed in several studies after sunitinib or bevacizumab [94], combining SA inhibitors with drugs blocking cell invasion or adhesion associated with VC should be worth examining.

## 6. Vasculogenic Mimicry and Endothelial Transdifferentiation

The first evidence of vasculogenic (or vascular) mimicry (VM, Figure 2) comes from studies on malignant melanoma, where fluid-conducting, matrix-rich channels or lacunae formed by aggressive tumor cells without the contribution of endothelial cells were observed [95]. Although initially widely debated, VM is now recognized as an important mechanism of vessel formation in cancer. In VM, tumor cells with multipotent, stem cell-like phenotypes acquire endothelial-like properties, form tubular networks, and secrete a dense matrix composed of collagen IV and VI, proteoglycans, heparan sulfate, and laminin, that contributes to the formation and stabilization of the structures. These channels are connected to the surrounding microcirculation at the tumor periphery and become perfused and functional.

VM is based on the plasticity of stem cell-like subpopulations of tumor cells that express cancer stem cell (CSC) markers such as CD133 [96], and involves different degrees of cell differentiation and outcomes. In patients with small cell lung cancer, a subset of circulating tumor cells (CTC) expressing vascular endothelial cadherin (VE-cadherin) and cytokeratins has been described. When implanted in immunocompromised mice, these cells retained the ability to form vessels expressing molecular markers of human tumors [97]. Tumor cells can fully transdifferentiate into ECs, but in most cases, VM cells still retain stem cell-like properties and markers [98,99,100].

VM is usually detected as vascular structures positive for periodic acid-Schiff (PAS), that recognizes glycoproteins, and negative for endothelial markers CD31/CD34 [101]. However, some authors refer to VM as CD31+/PAS+ vessels, implying vessels formed by fully transdifferentiated cancer cells. Consensus on the definition and markers of VM and endothelial transdifferentiation is still lacking and is urgently needed for proper analysis of this phenomenon, and to study the mechanisms involved and therapeutic approaches.

VM has been described in different tumor types, usually in highly invasive and advanced cancers, characterized by tumor cells with high plasticity and stem cell-like properties. A recent study identified melanoma-to-endothelial transdifferentiation as a potential mechanism contributing to melanoma dormancy in intravascular niches at metastatic organs [102]. Besides melanoma, VM and endothelial transdifferentiation have been identified in clinical and experimental samples of breast cancer, lung cancer, ovarian cancer, prostate cancer, hepatocellular carcinoma, glioblastoma, and colorectal cancer [103,104], often correlated with a more aggressive tumor phenotype and poor prognosis of patients [97,105,106].

### 6.1. Mechanisms of VM

As expected with a such a complex process, numerous molecular mechanisms contribute to VM. The process is induced by the hypoxic tumor microenvironment, and is sustained by the integration of a number of signaling pathways involved in the key aspects, including cancer stemness, response to hypoxia, vascular development, and extracellular matrix deposition and remodeling (reviewed in [103,104]).

Hypoxia is an important regulator of VM. Through HIF-1α-mediated control of different transcription factors, hypoxia regulates the phenotype and function of CSCs, affecting their self-renewal ability and differentiation potential. Among the most important pathways sustaining VM are VE-cadherin, EphA2, Nodal/Notch, and MMP2-mediated cleavage of laminin 5γ2 into 5γ2x and 5γ2′. Other players are VEGFR1, LOXL2 (lysyl oxidase-like 2) fibroblast growth factor receptor 2 (FGFR2), platelet-derived growth factor receptor beta (PDGFRβ) and the IL-8/CXCR2 axis, that have been considered potential therapeutic targets [48,103,106]. Recently, microRNAs (miRNAs) and long non-coding RNAs (lncRNAs) have been described involved in VM in different tumor types (reviewed in [104]).

### 6.2. VM and Resistance to Therapies

VM is a major cause of resistance to antiangiogenic therapies in solid tumors [86,107]. Disruption of tumor vessels by VEGF/VEGFR inhibitors generates a hypoxic microenvironment that promotes VM channel formation by the residual tumor cells to sustain recurrence. In experimental models of melanoma, VM occurred as a compensatory adaptation in tumors that were initially responsive to VEGF blockade, but not in VEGF refractory tumors [108]. In orthotopic glioblastoma models, vatalanib (PTK787, a VEGFR2 inhibitor) and bevacizumab (VEGF-targeting antibody) induced VM [109,110]. VM induced by bevacizumab in glioblastoma involved drug-induced autophagy in glioma stem cells [111]. VM was associated with high expression of HIF-1α [109] and upregulation of the IL-8/CXCR2 pathway (implicated in glioblastoma stemness), sustaining the formation of functional PAS-positive channels lined by CXCR2+ tumor cells. Impairing CXCR2 prevented VM in this setting [110].

Another VEGFR inhibitor, sunitinib, increased VM under hypoxic conditions in renal carcinoma models [112] and in triple-negative breast cancer cells through the overexpression of HIF-1α, VE-cadherin, and Twist1 [113,114].

VM has also been implicated in resistance to trastuzumab, a humanized antibody against the EGF receptor HER2, in breast cancer. CD31-negative and PAS-positive VM channels were higher in tumor samples from HER2-positive patients receiving trastuzumab than in tumors from untreated patients (or after chemotherapy alone), or tumors biopsied before treatment. In vitro, breast cancer cells resistant to trastuzumab showed a phenotypic switch towards stemness and the acquisition of vascular features [115].

Resistance to chemotherapy has also been related to VM. In experimental models of small cell lung cancer, VM was associated with a lower response to cisplatin, despite better distribution of the drug to the tumor [97].

### 6.3. Targeting VM

Agents targeting the multiple molecular effectors of VM have inhibitory properties on this process [104]. PD173074, an inhibitor of FGFR, inhibited VM in breast cancer [106]. Inhibitors of PDGF/PDGFR, including imatinib, were active on VM in triple-negative breast cancer [106] and melanoma [116]. Galunisertib (a selective inhibitor of TGF-βR1) inhibited VM in glioma through downregulation of VE-cadherin [117]. Other compounds targeting VM are histone deacetylase (HDAC) inhibitors in triple-negative breast cancer [118] and glioblastoma [119], the anti-helminthic drug niclosamide in oral cancer [120], and nicotinamide in aggressive melanoma [121].

In some cases, these inhibitors are not specific for VM, and are also active on other aspects of tumor progression including SA, as in the case of the inhibitor of 20-hydroxy eicosatetraenoic acid (20-HETE) synthesis, HET0016 [109], of inhibitors of dimethylarginine dimethylaminohydrolase (DDAH) involved in homeostatic control of nitric oxide [122], and of approaches based on the integrin-recognition sequence Arg-Gly-Asp (RGD), such as cilengitide [123].

Nanoparticle-based approaches have been proposed to target established VM, including cyclic RGD-functionalized nanoparticles [124], and liposomes incorporating chemotherapy and VM-targeting drugs [125,126]. The microtubule depolymerizing, vascular disrupting agents combretastatin A4 and DHPAC are another example of compounds that target already formed VM, rather than preventing it [127].

CVM-1118 (Foslinanib) is the first-in-class inhibitor of VM to enter clinical trials [128]. It is currently at Phase II. This small molecule downregulates stem cell-associated genes including Nodal, relevant for VM [103].

Since VM frequently emerges as an adaptive response to antiangiogenic therapy, a promising strategy would be to use a combination or sequential therapies to target VM and endothelial-dependent angiogenesis. A successful example is the use of the mammalian target of rapamycin(mTOR) inhibitor everolimus as second-line after treatment with sunitinib, which showed some efficacy in a model of renal carcinoma [112].

## 7. Conclusions

This review presented evidence that different types of tumor vascularization have an impact on tumor responses to antiangiogenic and antineoplastic therapy and can contribute to unsuccessful results in clinical trials and in the treatment of cancer patients [129]. Co-opted vessels, vasculogenic mimicry, or angiogenic vessels should be used to stratify patients for antiangiogenic therapies. This strategy has been successfully applied in colon carcinoma liver metastasis, where antiangiogenic treatment affected only lesions with SA and not those with co-opted vessels [130]. However, robust histological and molecular signatures to stratify patients on the basis of the type of vascularization are still needed. Several efforts have been made in this direction. Gene expression profiling at the single cell level across species and across tumors identified potential biomarkers of the endothelial cell tip phenotype, reflecting active SA [131]. A vascular microenvironment signature was associated retrospectively with response to bevacizumab [132].

The introduction of these analytical approaches will hopefully lead to the identification of markers selectively expressed on endothelial cells in the tumor vessels which, when exposed to the tumor microenvironment, acquire a phenotype different from endothelial cells in normal tissues [133]. This would pave the way to therapeutic strategies that selectively target vessels in tumors—independently of the modality of vessel formation—as direct therapeutic targets or to deliver drugs selectively to the tumor site.

Several potential targets have been identified, associated with tumor endothelial cells and stroma, and used to generate antibody-drug conjugates or CAR-T cell-based strategies for cancer therapy [133,134,135,136,137,138]. CD276 (B7-H3) is an appealing target. Besides being expressed by cancer cells, it is expressed by endothelial cells of both co-opted and angiogenic vessels in primary tumors and metastasis but is almost undetectable in normal tissues. This protein has a dual value, as an immune checkpoint and as a marker of tumor vessels. Anti-CD276-drug conjugate has been described as capable to selectively attack both the tumor and tumor-associated vessels [139]. The selection of the drug conjugate and antigen expression threshold in normal tissues will be critical for the success of therapies targeting tumor vessels.

The occurrence of the different forms of tumor vascularization depends on the properties of the tumor cells (e.g., plasticity of melanoma cells favors VM) but also of the organ affected by the primary tumor or metastasis (e.g., VC is often found in tumors or metastasis affecting the lungs and liver). Given the relevance of these vasculature structures in tumor response/resistance to therapies, preclinical studies should use suitable experimental tumors and metastasis in the proper organs (including orthotopic tumors and genetically engineered mouse models) to evaluate therapies in the correct microenvironmental context.

The optimization of antineoplastic therapies must also consider the importance of hypoxia as a master driver of all forms of tumor vascularization. Approaches aimed at promoting microenvironment normalization and re-education [140] should be used in combination with, or as maintenance therapy after, conventional antineoplastic therapies to avoid the generation of a hostile hypoxic environment and prevent microenvironment-mediated drug resistance.
